# Granulosa cell tumor of the ovary: a series of 6 cases

**DOI:** 10.11604/pamj.2024.47.58.38324

**Published:** 2024-02-11

**Authors:** Rafael Everton Assunção Ribeiro da Costa, Maria Clara Amorim Silva, Erlan Clayton Xavier Cavalcante, Rodrigo de Oliveira Castelo Branco, Cristiane Amaral dos Reis, Sabas Carlos Vieira

**Affiliations:** 1 Health Science Center, State University of Piauí, Teresina (PI), Brazil,; 2 Clinical Oncology, Oncocenter, Teresina (PI), Brazil,; 3 Tocogynecology, Oncocenter, Teresina (PI), Brazil

**Keywords:** Gynecological cancer, ovarian cancer, granulosa cell tumor

## Abstract

Granulosa cell tumor (GCT) is a rare ovarian malignancy that represents only 2-3% of all cases. There are two subtypes of GCT: juvenile/JGCT (5% of cases) and adult/AGCT (95% of cases). This study aimed to describe a series of 6 GCT cases. The 6 study patients were managed from June 2011 to November 2022 in a private oncology clinic located in Teresina (PI), Brazil. At diagnosis, the mean patient age was 47 years, and symptoms in 5 patients (83%) were pelvic pain and/or increased abdominal volume. The majority of the patients (N=4/67%) had no comorbidities or findings related to GCT on physical examination. The mean tumor size was 11 cm. Five (83%) tumors were stage Ia and one tumor (17%) was stage III. Regarding tumor subtype, 5 (83%) were AGCT and 1 (17%) was JGCT. Surgical treatment consisted of unilateral salpingo-ophorectomy in 2 patients (33%), total hysterectomy and bilateral salpingo-ophorectomy in 3 patients (50%), and cytoreduction (suboptimal) in 1 patient (17%). After a mean follow-up period of 62.7 months, 5 patients (83%) are still alive and free of disease. One (17%) died from disease progression after 126 months. In the current study, disease-free overall survival was 83%, in a mean follow-up period of 62.7 months.

## Introduction

Granulosa cell tumors (GCT) are the most common sex cord-stromal tumors of the ovary and are present in 70% of these cases. Nevertheless, these tumors are rare, accounting for only 2 to 3% of ovarian malignancies. Granulosa cell tumors may occur at any age, although only 5% of the cases occur before puberty. There are two subtypes of GCT: juvenile (JGCT) and adult granulosa cell tumors (AGCT). Adult granulosa cell tumor is the most common subtype (95% of cases). Despite being a less aggressive cancer, there are reports of metastatic GCT. The mainstay of treatment for GCT is surgery. However, adjuvant treatment with chemotherapy and radiotherapy may be considered, in cases of extraovarian disease, although there is still no consensus about these indications [1-3]. This study aimed to describe a series of 6 GCT cases.

## Methods

**Study design:** this is an observational case study.

**Setting:** a chart review of all gynecological cancer patients ever treated in a private Oncology clinic located in Teresina (PI), Brazil was carried out, in search of GCT cases.

**Participants:** a retrospective analysis of the medical charts of 6 ovarian GCT patients was conducted. These patients were managed from June 2011 to November 2022.

**Variables:** clinical data (age and symptoms at diagnosis, comorbid conditions and findings on physical examination), anatomic pathology data (size, staging, and tumor subtype), and data on surgical treatment (type of procedure, follow-up duration, and current patient situation) were collected.

**Data sources/measurement:** only patient medical charts of the healthcare unit were used as data sources. Data collection was performed, tabulated, and analyzed descriptively with the use of the Microsoft Excel program for Mac 2011.

**Bias:** biases were the sample size of the study and conduction of the study in a single healthcare center.

**Study size/inclusion and exclusion criteria:** a total of eight medical charts of GCT cases managed at the healthcare center since the time of its inauguration were found. Of these, 2 cases were excluded for containing clinical and anatomic pathology data on incomplete and/or lack of surgical treatment. All the remaining 6 cases that contained satisfactory data were included.

**Quantitative variables:** quantitative variables including age at diagnosis, tumor size, and follow-up duration were adopted.

**Statistical methods:** considering the sample size of this study, a descriptive analysis was used as the statistical method, showing absolute and relative numbers of the variables addressed.

**Ethical consideration:** this study is part of a project on oncology patients of the healthcare facility that managed the cases described in the study. In compliance with all the current research ethical guidelines in Brazil, the study was approved by the Institutional Review Board of the State University of Piauí, under technical opinion number 4.311.835.

## Results

As shown in Table 1, the mean age of study patients at the time of diagnosis was 47 years. Symptoms in five patients (83%) were pelvic pain and/or increased abdominal volume at diagnosis. The majority of patients (67%) had no comorbidities or any findings related to GCT of the ovary during physical examination. Table 2 shows the anatomic pathology characteristics of the tumors. The mean tumor size of the series was 11 cm. Five patients (83%) had stage I tumors, limited to one ovary with an intact ovarian capsule, and 1 patient (17%) had stage III tumors, with peritoneal metastases [4]. Concerning tumor subtype, 5 tumors (83%) were AGCT of the ovary and 1 tumor (17%) was a juvenile GCT. Table 3 shows the main surgical treatment for GCT. Two patients (33%) underwent unilateral salpingo-ophorectomy (USO), 3 (50%) underwent total abdominal hysterectomy (TAH) and bilateral salpingo-ophorectomy (BSO) and 1 (17%) underwent cytoreduction (suboptimal). After a mean follow-up period of 62.7 months, 5 patients (83%) are alive and free of disease and 1 patient (17%) died due to disease progression after 126 months of the diagnosis of ovarian GCT.

**Table 1:** clinical characterization of study patients

Patient	Age at diagnosis (years)	Symptomatology at diagnosis	Comorbidities	Physical exam
Case 1	13	Asymptomatic	None	Normal
Case 2	44	Pelvic pain and increased abdominal volume	SLE, SAH, and DM	Pelvic tumor
Case 3	63	Increased abdominal volume	SAH	Pelvic tumor
Case 4	65	Pelvic pain	None	Normal
Case 5	36	Pelvic pain	None	Normal
Case 6	59	Pelvic pain	None	Normal

SLE: systemic lupus erythematosus; SAH: systemic arterial hypertension; DM: diabetes mellitus

**Table 2:** anatomic pathological characterization of the tumors addressed

Patient	Tumor size (cm)	Stage	Subtype
Case 1	8	Ia	Juvenile granulosa cell tumor of the ovary
Case 2	16	Ia	Adult granulosa cell tumor of the ovary
Case 3	23	Ia	Adult granulosa cell tumor of the ovary
Case 4	5.8	Ia	Adult granulosa cell tumor of the ovary
Case 5	6.5	Ia	Adult granulosa cell tumor of the ovary
Case 6	6	III	Adult granulosa cell tumor of the ovary

**Table 3:** surgical procedures performed and follow-up of study patients

Patient	Surgical procedures	Follow-up duration (months)	Current situation (November 2022)
Case 1	USO, contralateral ovarian biopsy, and cytology of peritoneal lavage	58	Alive without disease
Case 2	TAH, BSO, pelvic lymphadenectomy and epiplectomy	60	Alive without disease
Case 3	TAH, BSO, lymph node sampling and epiplectomy	17	Alive without disease
Case 4	TAH, BSO, lymph node sampling, epiplectomy, and cytology of peritoneal lavage	51	Alive without disease
Case 5	USO, biopsy of the contralateral ovary, and cytology of peritoneal lavage	64	Alive without disease
Case 6	Suboptimal cytoreduction	126	Death from disease progression (August 2022)

USO: unilateral salpingo-ophorectomy; TAH: total abdominal hysterectomy; BSO: bilateral salpingo-ophorectomy

## Discussion

In the current study, 5 patients (83%) were alive after a mean follow-up period of 62.7 months. The literature shows that the majority of women diagnosed with GCT have a favorable prognosis, with a 5-year overall survival of about 97-98%. Nevertheless, it is a peculiarity of AGCT that there are more frequent late recurrences. Therefore, in these cases, early diagnosis, appropriate treatment, and a prolonged follow-up period are of importance. In this study, it was observed that 1 patient (17%) had a recurrence 23 months after the initial diagnosis, and was deceased due to disease progression at 103 months after recurrence [5].

**Figure 1:**
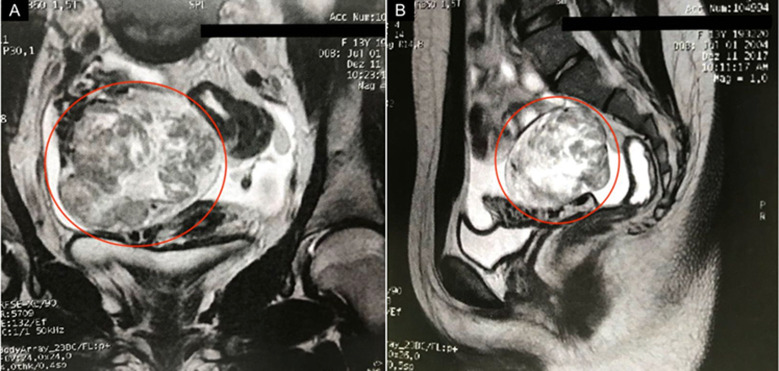
A, B) magnetic resonance imaging of the pelvis (case 1); the circled areas highlight the tumor region

**Figure 2:**
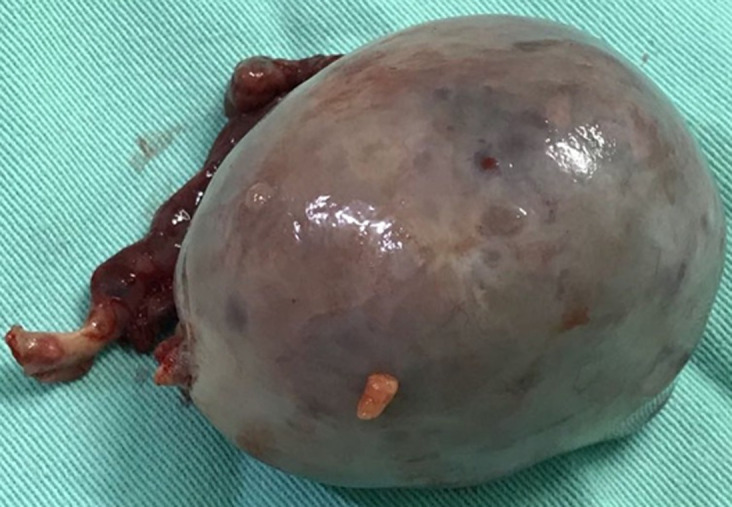
surgical specimen of the tumor (case 1)

Adult granulosa cell tumors patients are most commonly diagnosed at around 46 to 50 years of age. The clinical profile in these women is postmenopausal status and multiparity. In contrast, 80% of juvenile-type cases occur before the age of 20 years, and 45.5% of these cases occur before puberty. In the JGCT patient of the current study (case 1), the age at diagnosis was 13 years (after puberty) and the initial clinical picture was asymptomatic. In this case, the diagnosis was suspected after a pelvic ultrasound was performed at another service to investigate menstrual cycle alteration. Ultrasound detected a heterogenous right adnexal injury of around 8cm. The requested magnetic resonance imaging of the pelvis showed an 8cm right ovarian adnexal lesion with a neoplastic appearance (Figure 1A, B). Figure 2 below, the surgical specimen after surgical treatment. Immunohistochemistry (IHC) was positive for anti-cytokeratin, calretinin, Melan-A/Mart-1, and vimentin. Immunohistochemistry showed negativity for epithelial membrane antigen (EMA), CD99, inhibin, and WT-1. About 10% of the cases may be diagnosed during pregnancy. However, in our sample, there was no pregnant case. Regarding body mass index (BMI), the literature is consistent and describes that the disease is similarly distributed among normal-weight, overweight, or obese patients [2,6,7].

**Figure 3:**
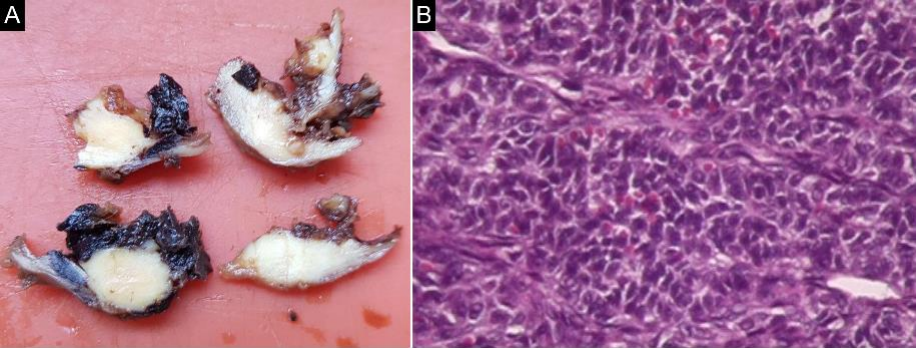
A) macroscopy of the surgical specimen in case 5; B): histopathology on hematoxylin-eosin showing GCT (200x magnification)

Most cases are diagnosed in stage 1 of the disease, especially owing to the symptomatic nature of the tumor. Therefore, early diagnosis is the main factor linked to a good prognosis [2]. For instance, the patient in case 5 presented with a unilateral 6.5 cm tumor without involvement of the ovarian capsule and with negative peritoneal fluid cytology. Macroscopy (Figure 3A) and hematoxylin-eosin histopathology (Figure 3B) confirmed the diagnosis of GCT. Immunohistochemistry was positive for FOXL2, calretinin, alpha-inhibin, and Wilms tumor gene product (WT1). Negative IHC for EMA and cytokeratin´s 40, 48, 50 and 50.6. Ki-67 positive (15%).

The most common symptoms are vaginal bleeding and a palpable abdominal mass. In our study, the most common symptom reported was pelvic pain (67%), followed by enlarged abdominal volume (33%). Regarding symptomatology, at the time of diagnosis, up to 20% of patients with AGCT are asymptomatic, compared to JGCT cases that are symptomatic in the majority of patients at the time of diagnosis. Pain and abdominal distension are the most common symptoms due to the mass effect. Some JGCT patients can also develop precocious puberty, including enlarged breasts, increased pubic hair and vaginal bleeding. An explanation for this finding is that the tumor is a hormone activator, with secretion mainly of estrogen. The association between virilization and testosterone secretion is uncommon, and it is found in only 10% of cases. It has been shown that cases associated with precocious puberty have a better prognosis, as a result of symptomatology and diagnosis at an early stage [4,6].

According to a previous study, in the majority of GCT patients, tumor size was 8 to 14cm [1]. In our sample, the mean tumor size was 11.5cm in AGCT and 8cm in JGCT. In 83% of the cases (5 patients) of this study, early-stage unilateral tumor occurred, with an intact ovarian capsule, corroborating previously published data, in which unilateral disease predominated [6,8].

**Figure 4:**
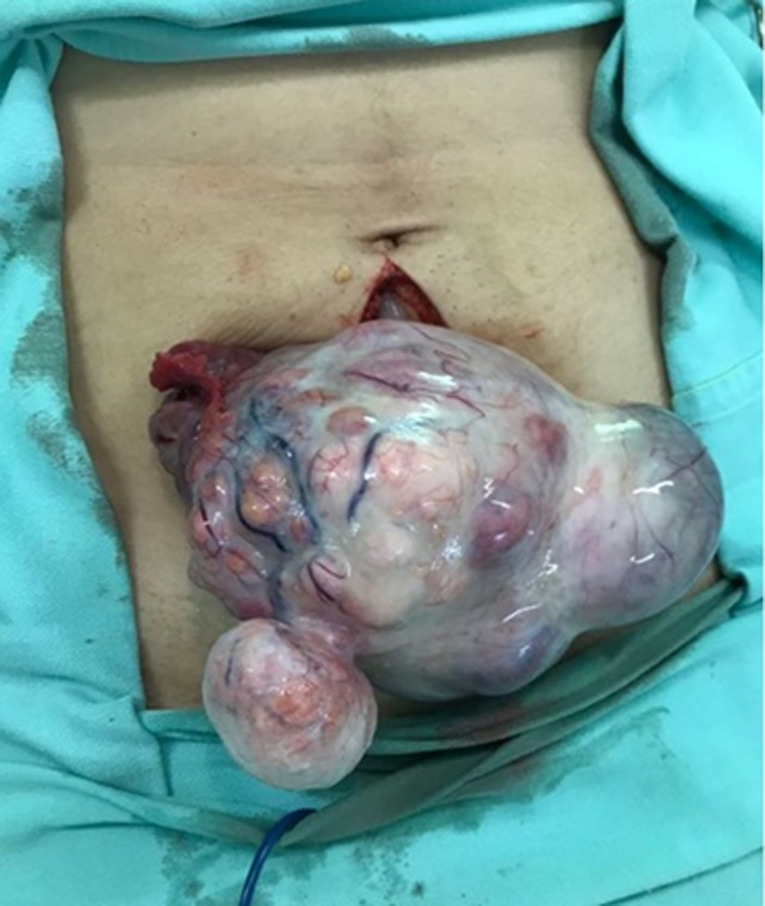
intraoperative total abdominal hysterectomy and bilateral salpingo-oophorectomy (case 2)

There is a significant association between GCT and endometrial disease. In a sample of 69 AGCT patients, around 26% of patients had evidence of endometrial disease, the majority of which was composed of hyperplasia without atypias. Despite the high prevalence rate, none of our patients was identified with endometrial alteration at the time of diagnosis and in the follow-up of cases treated with uterine preservation. The follow-up of postsurgical patients is performed with transvaginal ultrasound in cases of ovarian preservation. In the clinic that managed the study patients, hysteroscopy is only indicated when there is a change in endometrial thickness or persistence of abnormal uterine bleeding. The literature recommends follow-up of GCT patients by collecting a sample of endometrial tissue before proceeding with surgery to guide follow-up correctly [8]. In Figure 4 below, intraoperative total abdominal hysterectomy and bilateral salpingo-oophorectomy in case 2.

Concerning follow-up, the antimüllerian hormone (AMH) has a high sensitivity (92%) and specificity (81%) as a tumor marker both for primary and recurrent disease. In cases of detection for AGCT recurrence, the combination of AMH and inhibin B was more likely to be effective. Nevertheless, another tool with high indexes of sensitivity and specificity is the malignancy risk index (MRI), with about 84% sensitivity and 97% specificity that combines menopausal status, CA-125 status, and ultrasound findings. This tool is shown to be superior to the analysis of CA-125 alone. The neutrophil/lymphocyte ratio (NLR) is a new tool for the identification of various cancers and GCT patients have higher levels of NLR than controls [9,10]. In this study, inhibin B was measured in 4 patients (67%) (cases 1, 2, 4, and 5), and antimüllerian hormone was not measured in any case during follow-up. In cases where inhibin B was measured, high inhibin B levels were not detected in any case. In the study patients, inhibin B and antimüllerian hormone are less available due to the high costs involved.

The mainstay of treatment for AGCT and JGCT cases is surgery. If surgery is performed at an early stage, it offers high cure rates. Surgical procedures comprise total hysterectomy and bilateral salpingo-ophorectomy, with endometrial tissue analysis to exclude an association with endometrial alterations. The role of lymphadenectomy has not been fully established, especially in early-stage disease. However, patients with metastases and advanced stages of the disease should undergo complete cytoreduction, whenever possible. Fertility preservation may also be considered for early-stage disease, with unilateral oophorectomy, omentectomy, and peritoneal cavity analysis, with safety for stages 2B-3C [3,7,11]. Case 6 of this study underwent surgical treatment at an advanced tumor stage. Suboptimal cytoreduction was performed since a subcapsular hepatic lesion of 3.4cm, peritoneal nodular lesion of 1cm and external iliac chain lymph nodes of the pelvis measuring 1.6cm remained.

Chemotherapy has still not been well established for use in early-stage disease since it does not modify disease-free survival (DFS). Currently, although consolidation is lacking, it has been most commonly used in patients with disease recurrence, and metastatic and advanced disease. The most frequently used regimen is bleomycin, etoposide, and cisplatin. Concerning the use of adjuvant radiotherapy, data is still scarce. Nevertheless, an increase in the DFS rate has already been shown in patients receiving adjuvant radiotherapy compared to those who did not receive it (251x112 months), in a study using the resource in cases presenting with local isolated recurrence in advanced stages. There is a role for hormone treatment, either with aromatase inhibitors, progesterone, or gonadotropin releasing hormone (GnRH) agonists, for the treatment of metastatic disease, tumor recurrence, or unresectable tumors. Antiangiogenic or immunogenic drugs are useful in tumor recurrence, such as bevacizumab, a monoclonal antibody that blocks angiogenesis by inhibiting endothelial growth factor A (VEGF-A), which is expressed in excess in 94% of the patients with AGCT [9,12,13].

The diagnosis of recurrence is based on Histology, followed by imaging tests and increased tumor markers. Previous data have shown that recurrence occurs at around 10-64 months, with an interval of 48-57 months between the time of surgery and tumor recurrence, within 5 years of the initial diagnosis. The most commonly associated prognostic factors were staging, with about 40% odds of recurrence in stage 3, tumor size, and BMI. Diabetes mellitus and conservative surgery for the preservation of fertility were also associated with higher odds of recurrence. The latter was frequently performed in patients with JGCT, a group that is particularly at special risk of recurrence, especially if staging at the time of diagnosis is not appropriately performed. In contrast, the 5-year and 10-year DFS were considered excellent, with rates of 97% and 95%, respectively. Patients with advanced disease have an overall survival of 83-128 months. Following the data exposed, the patient with advanced disease in this study (case 6) was followed for 126 months and died from disease progression [10,13,14].

Limitations of our study were sample size (6 patients), lack of follow-up with the recommended tumor markers, and non-evaluation by hysteroscopy with endometrial biopsy of patients undergoing surgery with uterine preservation. A mean prolonged follow-up period is highlighted as relevant data, showing good survival in early-stage tumors.

## Conclusion

In the series analyzed, it was observed that most patients (83%) presented only general and non-specific symptoms at the time of diagnosis, such as pelvic pain and increased abdominal volume, showing the need for doctors and health professionals to be aware of the possibility of ovarian cancer in this context, even in patients before puberty, as observed in case 1 of the series. Among ovarian malignancies, GCT usually presents a good evolution, with the occurrence of extrapelvic disease being uncommon. However, when the disease spreads beyond the pelvis, the prognosis is quite poor, as shown in case 6 of the series, which died 126 months after diagnosis, highlighting the importance of early diagnosis and treatment in clinical conditions that may correspond to ovarian cancer, even in its rarer and less aggressive forms, such as GCT.

### What is known about this topic

- *Granulosa cell tumors are rare and represent only 2% to 3% of ovarian malignancies;*
- *Only 5% of granulosa cell tumors occur before puberty;*
- *The treatment of granulosa cell tumors is surgical, and adjuvant chemotherapy and radiotherapy may be considered in cases of extra ovarian disease*.

### What this study adds

- *Clinicopathological data of a rare tumor;*
- *Estimated survival in 6 cases of granulosa cell tumor;*
- *Detailed analysis of two unusual cases of granulosa cell tumors, one case of juvenile granulosa cell tumor and one case of extra-pelvic granulosa cell tumor*.
